# Editorial: Interplay of nutrition and genomics: Potential for improving performance and health of poultry

**DOI:** 10.3389/fphys.2022.1030995

**Published:** 2022-10-03

**Authors:** Faiz-ul Hassan, Mahmoud Alagawany, Rajesh Jha

**Affiliations:** ^1^ Institute of Animal and Dairy Sciences, University of Agriculture, Faisalabad, Pakistan; ^2^ Department of Poultry, Faculty of Agriculture, Zagazig University, Zagazig, Al Sharqia, Egypt; ^3^ Department of Human Nutrition, Food and Animal Sciences, University of Hawaii at Manoa, Honolulu, HI, United States

**Keywords:** nutrition, nutrigenomics, health, performance, poultry

Nutrigenomics is one of the most rapidly developing scientific fields that holds great promise for improving the health and performance of food-producing animals. Nutrigenomics begins a new era of working with nutrition and genetics, and gives us an insight into how nutrients interfere with an organism’s genetics and the resulting phenotypic response. It focuses on elucidating the effect of dietary nutrients on expression patterns of different genes and epigenetic modifications, such as DNA methylation and histone modifications (Fenech et al., 2011). The use of nutrients and nutraceuticals in poultry diets can improve expression of different genes related to immunity, metabolism, health, growth and antioxidant capacity (Alagawany et al., 2022). Nutritional programming during prenatal and postnatal life may have long-lasting consequences on the growth and health of birds and animals. Particularly during prenatal life, nutrients not only influence the developing embryo, but also affect the primary germ cells responsible for the next generation (Harrison, 2020). Therefore, exploring the effect of nutrients on genomic functions holds great promise to enhance the production performance and health of food animals.

Elucidation of epigenetic modifications controlled by nutrients is required to better understand the diet-gene interactions in avian species. Therefore, comprehensive studies are required to provide mechanistic insights into the nutritional regulation of gene expression through DNA methylation, histone modifications, and noncoding RNA interactions (Hassan et al., 2019). In addition, recent advances in high throughput techniques, such as whole-genome bisulfite sequencing, chromatin immune-precipitation sequencing, and global RNA-Sequencing, must be utilized for nutrigenomic studies in avian species.

This Research Topic covers the recent advances in nutritional and nutrigenomic interventions focused on improving health and performance, focusing on the discovery of diet-gene interactions in avian species. Various nutrients including phytonutrients have shown to affect different metabolic pathways in birds to modulate performance, immune response, and egg/meat quality. Phytochemicals in different herbs possess significant biological activities owing to their potential antioxidant and antimicrobial activities. These active compounds not only affect gut health but also enhance metabolic activity leading to enhanced nutrient digestibility and utilization (Hassan et al., 2020). Owing to their excellent biological activities of phytochemicals, their use as feed additives in poultry is increasing day by day to potentially modulate growth performance, gut health, immune response, and product quality. The biological activities of these phytochemicals mainly stem from their antioxidant and antimicrobial effects. Excellent antioxidant activities make them potential compounds to be used as therapeutic agents to scavenge free radicals (ROS) in the cell to mediate adverse effects of oxidative stress, which is a major challenge for bird’s health under extreme weather conditions. This is mainly attributed to the fact that higher production of ROS challenges cellular homeostasis and antioxidant defense leading to oxidative stress that subsequently affects vital physiological functions of the body (Mishra and Jha, 2019). The disruption of oxidative balance adversely affects the production performance and immune functions in birds since oxidative stress and inflammatory damage are multi-stage processes. The oxidative stress induced by higher ROS (H_2_O_2_) levels has shown to drastically affect the meat quality of broiler thigh muscle through mediating apoptosis, autophagy, and ROS/NF-κB signaling pathway (Yan et al., 2022). Furthermore, studies have shown that phytochemicals can also modulate key transcription factors involved in oxidative stress and inflammation, including nuclear factor (erythroid-derived 2)-like 2 (Nrf2) and nuclear factor kappa B (NF-κB) to regulate many downstream metabolic and signal transduction pathways (Lee et al., 2019). Regulation of different vital metabolic pathways through dietary supplementation of phytochemicals opens the horizon for nutrigenomic interventions to improve performance and health in poultry birds.

Dietary supplementation of herbs with rich antioxidant phytochemicals not only protects against ROS damage but also enhances cellular antioxidant defense, which can enhance the quality and shelf life of poultry meat. Herbal adaptogen consisting of well-known immune-boosting herbs *Ocimum sanctum*, *Withania somnifera*, and *Emblica officinalis* showed positive effects on growth performance and feed conversion ratio (FCR) in heat-stressed broilers (Greene et al.). Supplementation of this combination of herbs also modulated the amino acid profile, pH, color, and quality of breast meat. This indicates that potent antioxidant activities of phytochemicals of these herbs alleviated adverse effects of ROS in birds while improving immune functions, meat yield and quality (Lee et al., 2019). Moreover, these phytochemicals can also be used to potentially enrich the meat with antioxidants with subsequent enhancement in shelf life and health-promoting effects. Dietary supplementation of different levels of garlic straw powder showed potential improvement in meat quality and antioxidant capacity of yellow-feathered broilers without affecting growth performance and intestinal mucosal morphology (Liao et al.).

The major stress alleviating effect of dietary phytochemicals is mediated by increasing the endogenous antioxidant enzymes while decreasing oxidant enzymes. For example, Alfalfa (Medicago sativa Linn)-mixed silage fermentation material enhanced the glutathione content in chest and leg muscles and serum superoxide dismutase (SOD) activity while reducing the muscle malondialdehyde content in Lande geese (Li et al.). In addition, it also substantially increased the serum concentrations of triglycerides, total cholesterol, urea, and aspartate aminotransferase, consistent with good liver and kidney function. This is mainly mediated by enhancing the expression of genes meant for producing antioxidant proteins through multifaceted pathways. For example, dietary supplementation of 6% ramie (*Boehmeria nivea*) powder promoted the antioxidative capacity of the ducks by increasing the serum activities of SOD and glutathione as well as the mRNA expressions of glutathione peroxidase (GSH-Px) in the breast meat and SOD in the leg meat (Lin et al.). Similarly, dietary inclusion of 3% ramie significantly increased the activities of liver SOD and GSH-Px in laying hens. However, the addition of 3%∼6% ramie powder significantly increased the villus height of jejunum and villus height/crypt depth of ileum, revealing desirable effect on intestinal development of layers (Wang et al.).

Phytogenic feed additives have been largely exploited in poultry feeds to modulate gastrointestinal functions and health, and their implications on the birds’ systemic health and welfare, the production efficiency of flocks, food safety, and environmental impact (Abdelli et al., 2021). Keeping in view of the potential of phytochemicals to modulate gastrointestinal functions and gut health in birds (Biagini et al., 2022), many feed ingredients with functional compounds have been evaluated in poultry feed to exploit the synergistic effects of nutrients and phytochemicals. For example, dietary inclusion of ramie (*Boehmeria nivea*) powder at various levels has shown to affect growth and health status in Linwu ducks, indicating 6% as an optimum inclusion level for better growth performance (Lin et al.). Similarly, dietary inclusion of ramie powder in the diet of laying hens also affected the egg composition, as the addition of 6% ramie significantly increased total omega-3 polyunsaturated fatty acids and phenylalanine in egg yolk (Wang et al.).

Inclusion of *Artemia argyi* (1%) in poultry feed enhanced the antioxidant capacity of laying hens through increasing T-SOD and CAT activities, as well as GSH-Px contents in the liver. However, the dietary supplementation of 3% A. argyi substantially increased the serum and liver MDA contents and adversely affected the intestinal morphology by increasing duodenal crypt depth (Chen et al.).

In addition to antioxidant activity and immunogenic effects, phytochemicals have also shown to increase nutrient digestibility and metabolism in poultry birds. In order to formulate high density diets, high fat levels are used which can put burden on liver and might lead to oxidative stress and fatty liver syndrome. Phytochemicals can also help in these conditions as certain compounds like phospholipids can improve liver function and alleviate oxidative stress and liver damage. Soy lecithin is a phospholipid and, being the major component of cell membranes, plays a key role in cell repair and liver health. Long-term dietary supplementation of soy lecithin reduced the MDA (product of lipid peroxidation in the liver) content while increasing the antioxidant capacity (Total antioxidant capacity, SOD, and GSH-Px contents) of the liver in laying hens (Hu et al.). These findings indicated that long-term dietary lecithin supplementation can enhance the blood and liver lipid contents in laying hens and also improve the antioxidant capacity of the liver ensuring liver health. Overall this research topic has provided inisghts about the modulation of growth, metabolism, antioxidant status and immune response using different dietary nutrients and phytochemicals in poultry. It is clearly evident that nutrigenomics will serve as a new tool for nutritional research in addressing the issues related with poultry production particularly bird’s health, oxidative stress and growth performance. In future, innovations in nutritional interventions with aid of various molecular technologies will help to better understand the nutrient gene interactions ultimately leading to find more refined and sustainable methods for managing poultry production. Nutritional manipulation for the targeted modulation of the specific genes seems quite possible in near future to get the desired performance in terms of better health and performance (Zhao et al., 1995; Salami et al., 2015).
